# *Ex-vivo* Alzheimer’s disease brain tissue investigation: a multiscale approach using 1060-nm swept source optical coherence tomography for a direct correlation to histology

**DOI:** 10.1117/1.NPh.7.3.035004

**Published:** 2020-08-21

**Authors:** Antonia Lichtenegger, Johanna Gesperger, Michael Niederleithner, Laurin Ginner, Adelheid Woehrer, Wolfgang Drexler, Bernhard Baumann, Rainer A. Leitgeb, Matthias Salas

**Affiliations:** a Medical University of Vienna, Center for Medical Physics and Biomedical Engineering, Vienna, Austria; b Medical University of Vienna, Division of Neuropathology and Neurochemistry, Department of Neurology, Vienna, Austria; c AIT Austrian Institute of Technology GmbH, Vienna, Austria; d Medical University of Vienna, Christian Doppler Laboratory for Innovative Optical Imaging and its Translation to Medicine, Vienna, Austria

**Keywords:** Alzheimer’s disease, neuroimaging, amyloid-beta plaques, optical coherence tomography, histology

## Abstract

**Significance:** Amyloid-beta (A-β) plaques are pathological protein deposits formed in the brain of Alzheimer’s disease (AD) patients upon disease progression. Further research is needed to elucidate the complex underlying mechanisms involved in their formation using label-free, tissue preserving, and volumetric techniques.

**Aim:** The aim is to achieve a one-to-one correlation of optical coherence tomography (OCT) data to histological micrographs of brain tissue using 1060-nm swept source OCT.

**Approach:** A-β plaques were investigated in *ex-vivo* AD brain tissue using OCT with the capability of switching between two magnifications. For the exact correlation to histology, a 3D-printed tool was designed to generate samples with parallel flat surfaces. Large field-of-view (FoV) and sequentially high-resolution volumes at different locations were acquired. The large FoV served to align the OCT to histology images; the high-resolution images were used to visualize fine details.

**Results:** The instrument and the presented method enabled an accurate correlation of histological micrographs with OCT data. A-β plaques were identified as hyperscattering features in both FoV OCT modalities. The plaques identified in volumetric OCT data were in good agreement with immunohistochemically derived micrographs.

**Conclusion:** OCT combined with the 3D-printed tool is a promising approach for label-free, nondestructive, volumetric, and fast tissue analysis.

## 1 Introduction

Alzheimer’s disease (AD) is the most common form of dementia worldwide. In 2019, 5.8 million people in the United States were suffering from AD. The numbers are increasing in our aging society, and there is still no cure available.^1^ Our society is facing a considerable social and financial burden due to severe cognitive impairment of affected patients. Ultimately, they are dependent on care-giving.^1^ On a cellular level, AD is characterized by the degeneration of neurons and the formation of intracellular neurofibrillary tangles composed of tau protein and extracellular plaques composed of amyloid-beta (A-β) protein.^2^^,^^3^ The definite diagnosis of the disease can only be done postmortem by histologic analyses of different regions of the cerebral cortex. Using different immunohistochemical and molecular methods, the presence of A-β plaques and tau protein tangles has to be confirmed.^4^ A-β plaques are in the range of 10 to 200  μm in diameter and have been investigated using light and fluorescence microscopy as well as Raman spectroscopy.^2^^,^^3^^,^^5^^–^^8^ For these imaging techniques, processing steps such as sectioning and molecular labeling of the tissue are required. Further research is urgently needed to fully understand the complex underlying mechanisms involved in AD and the formation of these plaques using a label-free, tissue preserving, and volumetric technique.^9^

Optical coherence tomography (OCT) is a nondestructive, label-free, and three-dimensional (3-D) imaging modality used to investigate anatomical features on a micrometer scale. The contrast in OCT images is based on the intrinsic scattering of light within the tissue.^10^ Using near-infrared light sources, micrometer resolutions and millimeter penetration depths were reported for brain imaging.^11^ OCT or OCT-based microscopy (OCM) has shown to be a promising tool for *ex-vivo* brain investigations.^12^^–^^14^ Studies using OCT for imaging *ex-vivo* human brain samples have shown that intensity-based OCT images can visualize tissue morphology and microstructure comparable to conventional histology.^15^^–^^17^ Further, OCT has been used to image and analyze A-β plaques nondestructively and label-free in both murine and human brains. In OCT images, the contrast is based on the inherent hyperscattering properties of these A-β plaques.^18^^–^^23^ First, in 2012, A-β plaques were investigated using a Bessel beam illumination OCM setup operating at 800 nm.^18^ A polarization sensitive (PS)-OCM setup at 840 nm was later utilized to identify plaques in postmortem brain tissue based on their intrinsic birefringence.^19^ Recently, Gesperger et al.^23^ conducted a study using a commercial PS-OCT setup to categorize A-β plaques depending on their inherent intensity and PS signal. Using visible light OCT, A-β plaques in murine and human brain tissue down to a diameter of 10  μm were visualized.^20^^,^^21^^,^^24^ However, all of these studies still lacked an exact one-to-one correlation to histology, which remains a general challenge in the field of *ex-vivo* OCT imaging.

Histology allows for a highly detailed understanding of the investigated tissue and is still the gold standard technique for analyzing AD-affected *ex-vivo* brain tissue today.^4^ The extracellular protein accumulations show different morphologies and can be divided into neuritic and diffuse plaques.^25^ In histology, neuritic plaques can specifically be labeled and visualized using, for example, Congo red staining.^26^ For conventional histologic analyses, tissue fixation, sectioning, and staining are required to achieve proper image contrast in micrographs. Using histology, cellular structures can be investigated at a molecular level.^27^ Drawbacks of histology are that it is time consuming and morphological alterations and tissue shrinkage is introduced during the workup.^28^ Ideally, brain tissue could be investigated using a tissue preserving, high resolution, 3-D imaging method, and findings would be confirmed by histology in a direct one-to-one correlation in which specific structures and landmarks are identified. This gained information could then be used for further brain-related pathological studies.

In this paper, we present the investigation of A-β plaques in *ex-vivo* human brain tissue samples using a 1060-nm swept source OCT (SS-OCT) setup. The system provided two fields-of-view (FoVs) and imaging with two transverse resolutions. A 3D-printed tool was developed to enable a direct correlation of the OCT results to histology. The presented work is a step toward the direction of nondestructive *ex-vivo* tissue analysis using a label-free optical imaging method, which can be directly correlated to histology, the current gold standard technique.

## 2 Methods

### 2.1 Swept-Source OCT Setup with Two Field-of-View Modalities

**Fig. 1:**
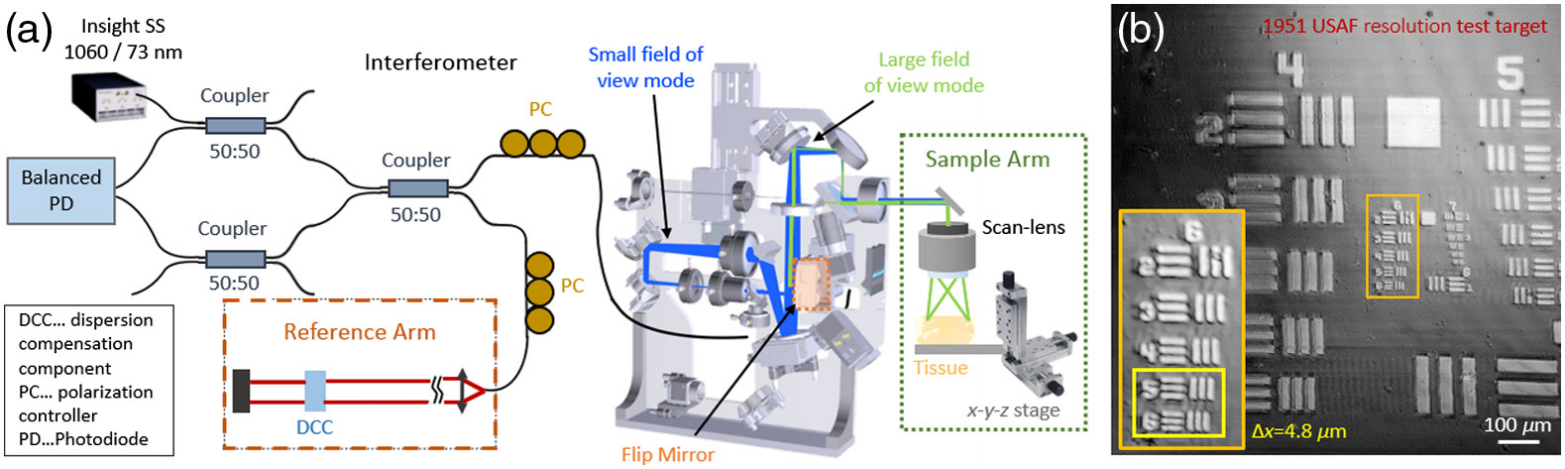
The SS-OCT setup and the lateral resolution measurement for the small FoV. (a) Schematic drawing of the SS-OCT setup with the two light paths for the large FoV, indicated by the green color, and the small FoV, indicated by the blue color, modes, respectively. The flip mirror for the small FoV imaging is indicated by orange. (b) The resolution test target measured with the small FoV. The sixth group and fifth/sixth element can be resolved, corresponding to a resolution of 4.92 and 4.38  μm, respectively.

A modified ophthalmic SS-OCT instrument offering the choice of sequentially using two FoV modalities was utilized to image brain tissue in a microscopic scheme; see Fig. 1(a).^29^ The light source used for this measurements was an Insight swept source at 1060 nm with 73 nm bandwidth and an A-scan rate of 100 kHz. The axial resolution was measured to be 8  μm in air, which corresponds to 5.9  μm in brain tissue, assuming a group refractive index of 1.36.^30^

**Fig. 2:**
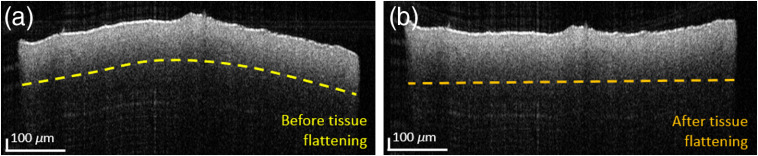
The tissue flattening of the control brain. (a) B-scan image before tissue flattening. (b) The control brain B-scan image after tissue flattening.

A lens (ThorLabs, AC254-30-C, 30-mm focal length) was integrated at the conjugated position of the scanners in the sample arm. With the large FoV, an area of 15  mm×15  mm was covered and a lateral resolution of 48  μm was calculated. In the small FoV mode, an imaging range of 1  mm×1  mm was covered and the lateral resolution improved to 4.8  μm. A resolution target was imaged to measure the transverse resolutions; see Fig. 1(b). By switching an automatic flip mirror [indicated in orange in Fig. 1(a)], the large FoV light beam diameter, indicated in green, was expanded at the entrance pupil of the lens, from 0.8 to 8 mm, indicated in blue, for imaging with the high lateral resolution mode.^29^ For the acquisition, volumes with a sampling of 1000×1000×4096  pixels were acquired in 10 s. Standard OCT postprocessing steps were applied to retrieve the intensity volumes. To improve the signal-to-noise ratio in the OCT intensity images, a 3-D average filter with an isotropic pixel size of three was applied. Due to the distortions introduced by the optics in the large FOV mode, tissue flattening had to be applied. These distortions arise from path length differences of the beams in central and peripheral regions. A paper surface was imaged as a flat reference target, and the images of the brain tissue were multiplied with the same x-y map of z-offset factors. Figure 2 shows the B-scan images of a control brain before and after applying the tissue flattening. Using the 1060-nm SS-OCT setup, an imaging range of ∼∼600  μm in brain tissue was achieved.

Using the OCT volumetric data, averaged and maximum intensity en-face projections over various depths were generated for a direct comparison with histology images.

### 2.2 Tissue Preparation and OCT Imaging

**Fig. 3:**
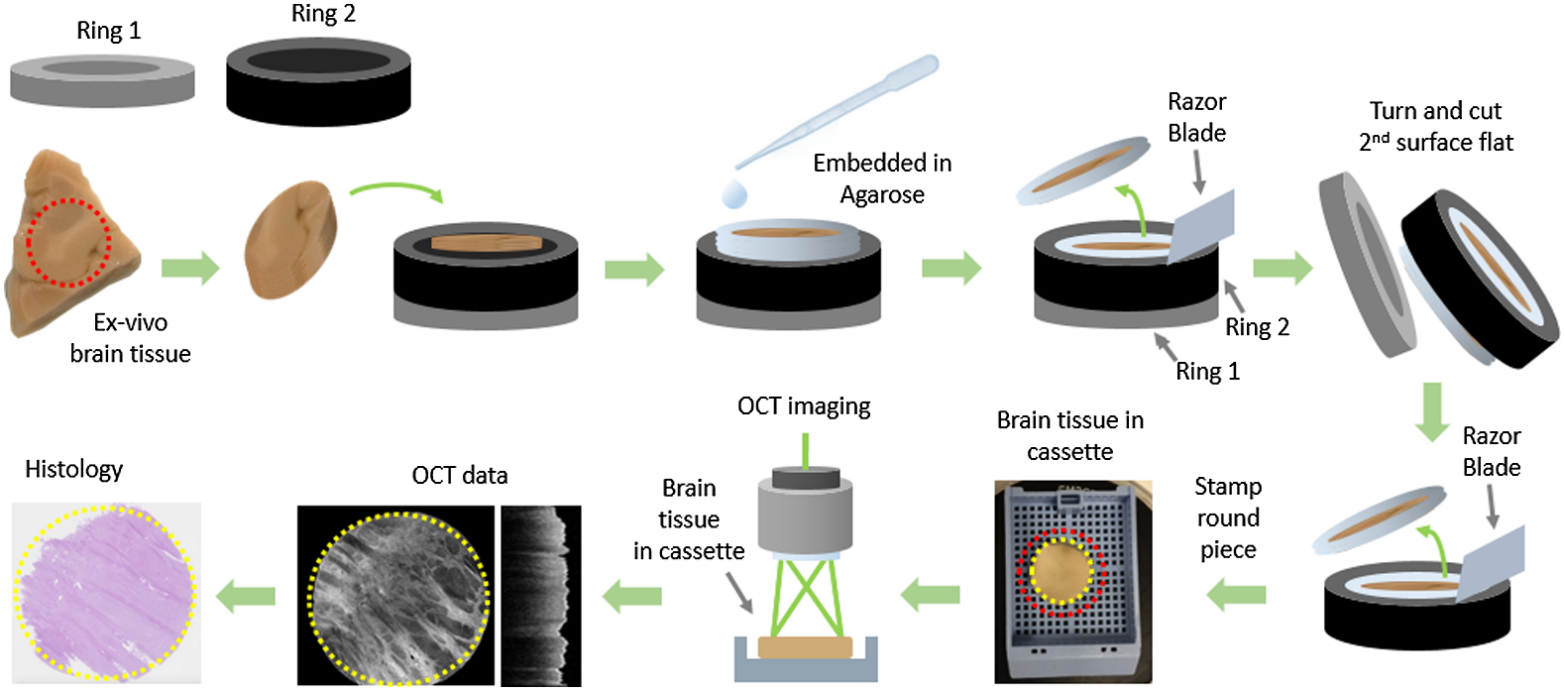
The brain tissue processing and imaging steps. First the formalin-fixed brain tissue was shaped using the 3D-printed tool. Next, the OCT measurements were performed. The OCT data were processed and flattened before en-face projections were generated. The tissue was embedded, sliced, and stained to gain histology micrographs.

Formalin-fixed, unlabeled brain tissue samples of human patients diagnosed with end-stage AD and non-neoplastic cerebral tissue such as the cortex and the pons as control cases were investigated. Human brain samples were provided by the Neurobiobank of the Medical University of Vienna (ethics approval number 396-2011). The samples were taken from the frontal cortex and the pons. An overview of the steps for the *ex-vivo* brain tissue imaging is shown in Fig. 3. First, to achieve parallel and straight tissue surfaces, a 3D-printed tool consisting of two plane rings (each had an inner diameter of 35 mm) of three and five millimeters in height was designed. This allowed for achieving perfectly flat surfaces for OCT imaging and histological correlation as described below. A round piece of brain, with a diameter of 25 mm, was punched from a large (in the range of 10  cm×10  cm) sample. This piece was put into the two rings being placed on top of each other and embedded in 5% agarose gel. The excess tissue reaching out of ring 1 was removed using a razor blade, which was guided carefully over the plane surface of the ring.

Then, the lower ring (ring 1) was removed, and ring 2 was flipped to cut the second surface using the razor blade, thus obtaining two parallel, flat surfaces. Finally, a smaller round piece (15 mm in diameter) was cut out, resulting in an agarose-free tissue sample. This sample was imaged in a standard histology cassette. By imaging the sample in the cassette, additional movement of the tissue was avoided. OCT measurements were performed, and immediately after imaging, the samples were processed for histologic workup. Care was taken that the orientation of the sample was maintained, and no movement artifacts were introduced throughout the whole process.

### 2.3 Histology

The brain samples were embedded in paraffin and sectioned into 3-μm slices using a microtome. Immunohistochemical staining against A-β [Dako Beta-Amyloid 1:50 (M0872, Clone 6F/3D), Detection system Dako EnVision] was performed to confirm the presence of A-β accumulations in AD and control brain samples. For all of these sections, hematoxylin was used as a nuclear counter staining. In addition, some of the sections were stained using Congo red staining to confirm the presence of neuritic A-β plaques. Congo red images were counterstained using hematoxylin to gain a general overview of the tissue morphology. For the pons histology, standard hematoxylin and eosin (H&E) staining was conducted. Digitized micrographs were acquired with a slide scanner (C9600-12, Hamamatsu). The micrographs were acquired using a 40× commercial objective lens providing a transverse resolution of 0.23  μm. To simplify the process of direct comparison of OCT with histology, multiple consecutive histological sections were performed. No A-β plaques were found in OCT or histology data of the control brain; see Fig. 2.

### 2.4 Correlation of OCT and Histology Data

**Fig. 4:**
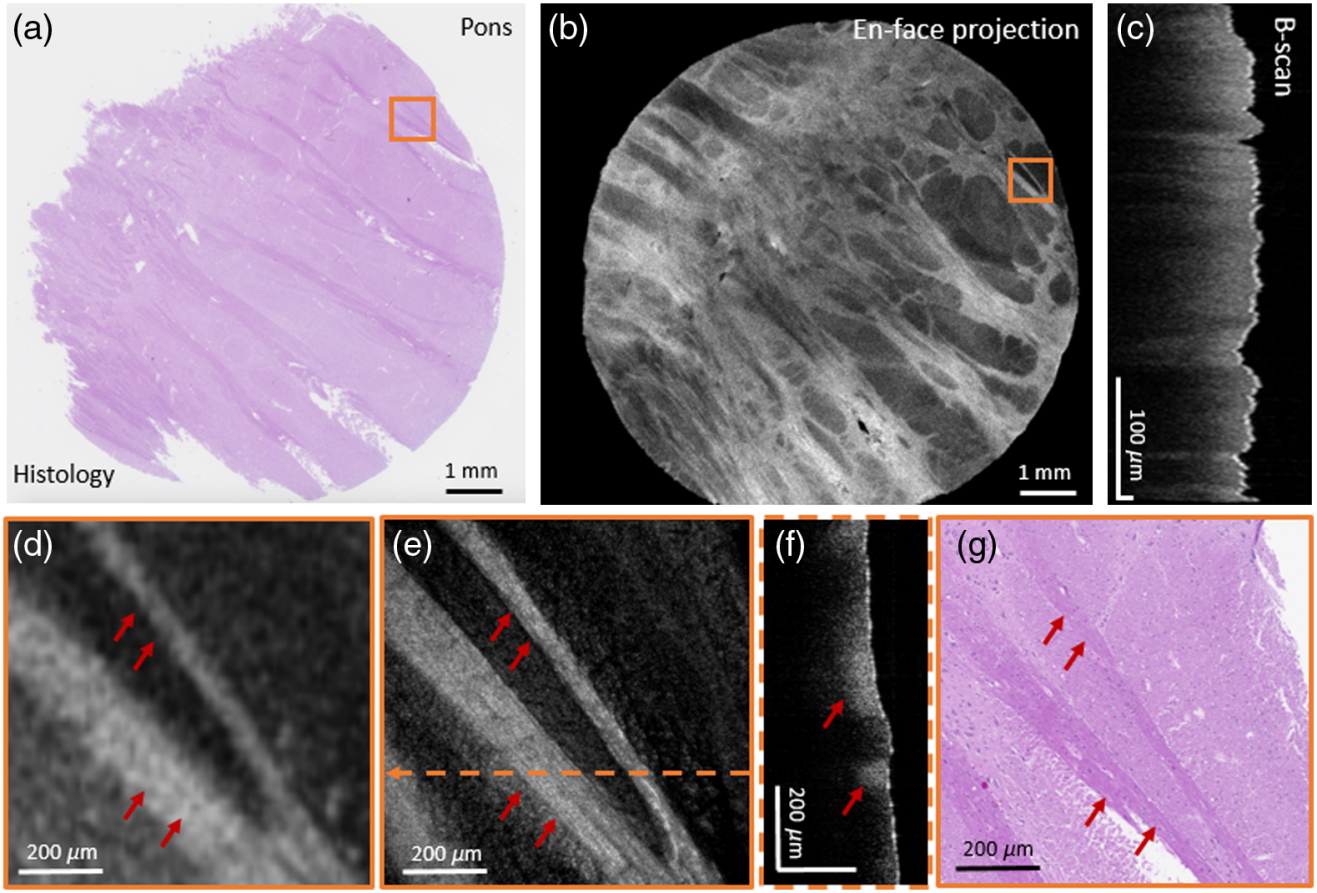
Pons tissue imaging. (a) H&E-stained histological micrograph. (b) Averaged en-face projection over 600  μm. (c) The corresponding B-scan image of the large FoV. (d) Zoom-in of the indicated orange square in the large FoV mode. (e) High-resolution image at the coregistered position. (f) Corresponding B-scan image (e) in high-resolution mode. (g) A zoom-in of the corresponding histology image. Fiber structure, typically found in pons tissue, is indicated by red arrows in OCT and histology images.

**Fig. 5:**
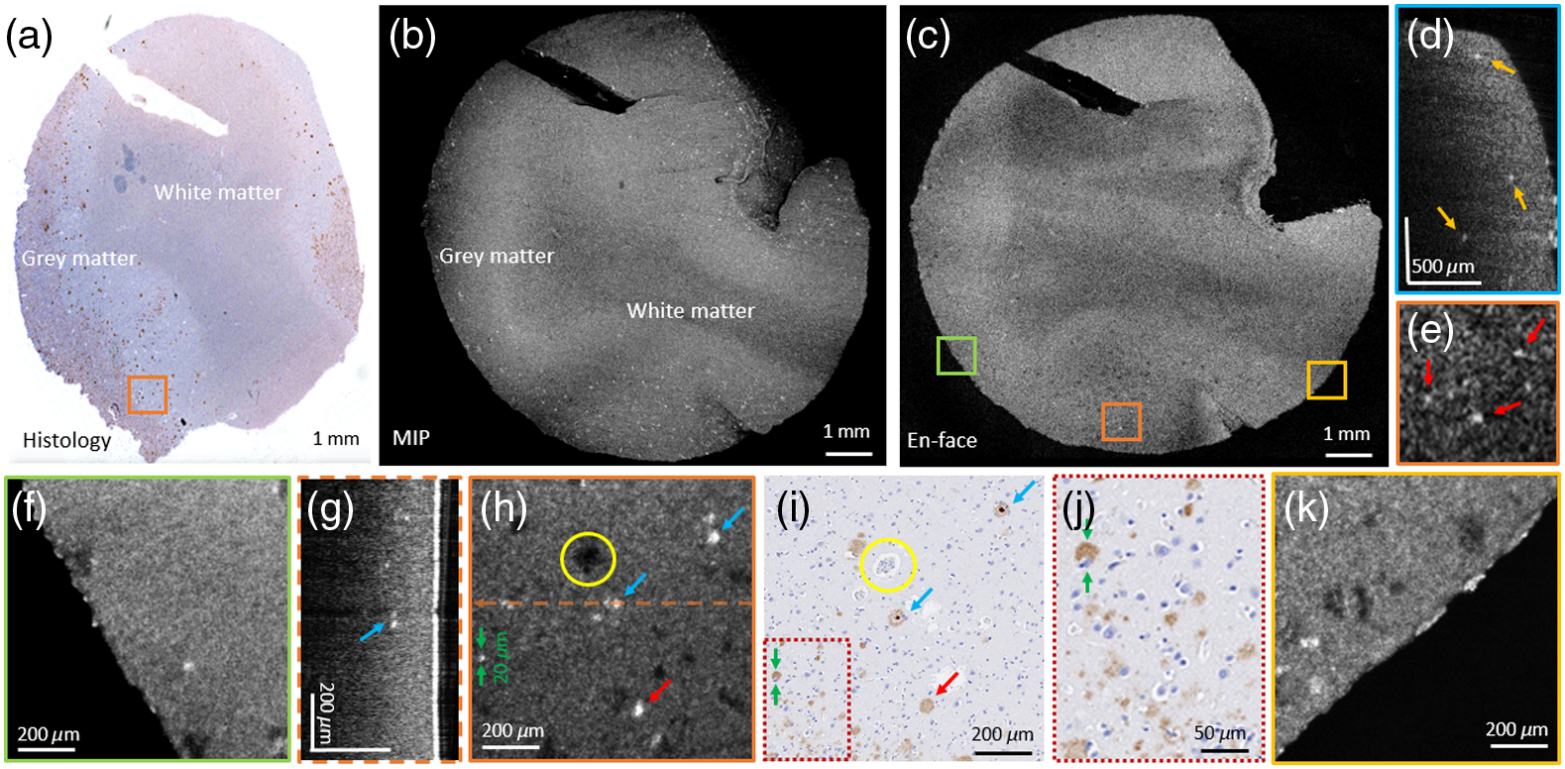
Imaging of A-β plaques using OCT and conventional histology. (a) Immunohistochemically stained brain section. (b) Large FoV OCT MIP over 600  μm. (c) Averaged en-face projection over 3  μm at the same depth position as in the histology image (a). (d) Cropped B-scan image showing hyperscattering A-β plaques throughout the whole imaging depth, indicated by yellow arrows. (e) Zoom-in of the region indicated by the orange square. (g) High-resolution B-scan image at the indicated position in (h). (f), (h), and (k) ROI including plaques, imaged with the high-resolution OCT mode. (i) Immunohistochemically stained section of the same depth position as shown in (h). (j) Zoom-in of the region indicated by the red, dashed square. The plaques can be identified as brownish accumulations in histological images. In all OCT images, the plaques can be identified as highly scattering regions. Selected A-β plaques are marked using red (diffuse plaques) and blue (dense plaques) arrows, and a vessel structure is labeled using a yellow circle.

**Fig. 6:**
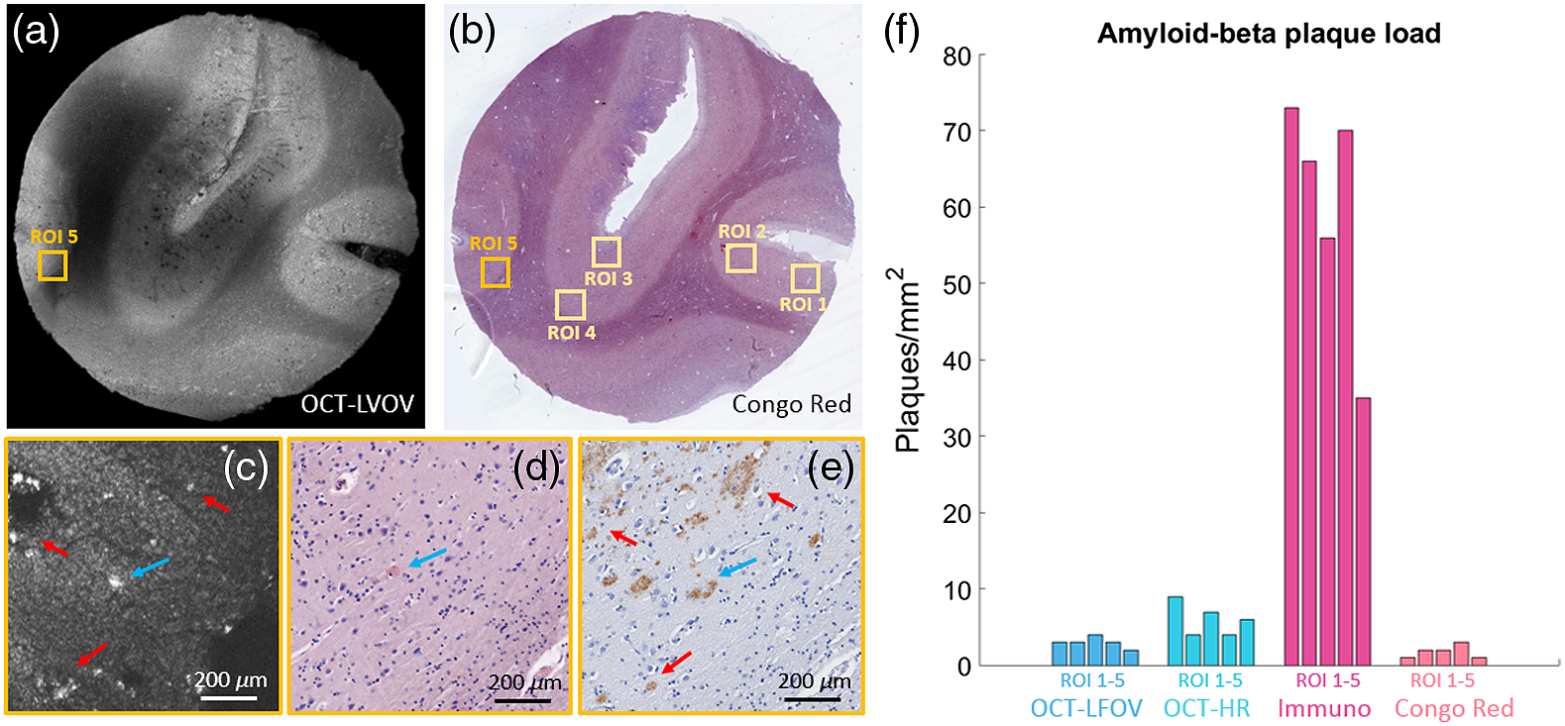
Evaluation of the A-β plaque load in OCT and histology. (a) Large FoV OCT (OCT-LFOV) averaged intensity projection over 600  μm. (b) The corresponding Congo red-stained histological section including the five evaluated ROIs. (c) High-resolution OCT (OCT-HR) intensity image taken at position indicated by a dark orange square in (a) and (b). (d) Zoom-in of the Congo red-stained section in (b). (e) Corresponding immunohistochemical stained section, showing a high density of A-β plaques (brownish accumulations). A neurtic plaque is indicated by a blue arrow, and diffuse plaques are marked with red arrows. (f) Bar plots showing the evaluated plaque load in the five ROIs in OCT-LFOV, OCT-HR images, and the immunohistochemical (Immuno) and the Congo red-stained sections.

First, for the correlation of OCT and histology data, the large FoV acquired by OCT was used. The OCT images were orientated using the tissue morphology of the histology micrographs. For example, in the AD brain tissue, shown in Figs. 5(a)–5(c), the crack on the upper left corner was used and for the pons the fiber structures; see Fig. 4. Utilizing again distinct tissue features, for example, the highly scattering plaques, vessel, or fiber structures, the depth position of the histology section was located in the OCT volumes. To align image data from OCT and histology, landmarks visible in both modalities such as A-β plaques were used. To improve and speed up the registration process, we used a Matlab algorithm to further align the histology micrographs to the large FoV OCT images.^31^ The automatic image registration could only be performed on micrograph images, where no substantial part of the tissue was lost during the histology process; see Fig. 6. The high-resolution OCT images acquired using the small FoV, were directly correlated to the large FoV images and then compared with the high-resolution micrographs from histology.

For an initial experiment, brain tissue obtained from the pons region was used to evaluate the direct correlation of histology to OCT images; see Figs. 4(a) and 4(b), respectively. Figure 4(b) shows an averaged en-face projection over 600  μm, and Figs. 4(c) shows the corresponding B-scan image. A zoom-in of the large FoV image, indicated with an orange square in Fig. 4(b), is shown in Fig. 4(d). Using the high-resolution mode, Fig. 4(e), the perfect colocalization of the two sequential imaging modalities is shown. Figure 4(f) shows a B-scan in high-resolution mode. Figure 4(g) shows a zoom-in of the histological micrograph. Fiber structures typically found in pons tissue are indicated by red arrows and can be seen as highly scattering regions in the OCT images.

To quantitatively compare histology and OCT results, the plaque load in five regions of interest (ROIs) (each 1  mm×1  mm), in an OCT intensity en-face projection over 3  μm and the corresponding histology image, was manually evaluated using Fiji.^32^ In OCT images, plaques were identified based on their appearance as volumetric clusters of hyperscattering pixels. For the histological sections, a neuropathologist assisted with the identification and classification of the various amyloid-beta deposits. The plaque load in the high resolution, large FoV OCT data and in the immunohistochemical and Congo red staining corresponding micrograph areas was evaluated.

## 3 Results

A-β plaques were identified as highly scattering features within the gray matter in OCT images and as brownish accumulations in the immunohistochemically stained histology images; see Fig. 5. Figures 5(a)–5(c) show a direct comparison of the immunohistochemically stained histology image with a large FoV OCT maximum intensity projection (MIP) over 600  μm (b) and an average intensity en-face projection over 3  μm (c), respectively. Figure 5(d) shows a cropped B-scan image acquired with the large FoV mode. Hyperscattering A-β plaques are visible throughout the whole imaging depth, indicated by yellow arrows. The en-face projection in Fig. 5(c) was taken at the same depth position as the histology section; see Fig. 5(a).

Figure 5(e) shows a zoom-in of the large FoV image (c). The high-resolution OCT intensity en-face images, taken at the positions marked with colored boxed in Fig. 5(c), are shown in Figs. 5(f), 5(h), and 5(k), respectively. In Fig. 5(g), a B-scan image of the high-resolution mode is shown. Figure 5(i) shows the corresponding immunohistochemically stained section of Fig. 5(h), where the same plaques in both images are marked by red (diffuse plaques) and blue (dens plaques) arrows. A zoom-in of Fig. 5(i) is shown in Fig. 5(j). In addition, a vessel structure could be observed and was marked by a yellow circle.

Another cortex region in an AD-affected brain tissue was investigated, and the averaged intensity en-face projection over 600  μm of the large FoV OCT image is shown in Fig. 6(a). The corresponding Congo red-stained histological section is shown in Fig. 6(b). The five ROIs are indicated by orange squares. The high-resolution OCT image taken at the position indicated by the dark orange square in Fig. 6(a) is shown in Fig. 6(c). The corresponding zoom-in of the Congo red-stained micrograph is shown in Fig. 6(d), and the immunohistochemical stained image is shown in Fig. 6(e). A neuritic plaque is indicated by a blue arrow, and diffuse plaques are marked with red arrows. The plaque load was manually evaluated in five ROIs (1  mm×1  mm). In the high-resolution OCT images, a higher plaque load ($6.0\pm 2.1\;\text{plaques}\;\mathrm{per}\;{\mathrm{mm}}^{2}$, median±std) was observed compared with the large FoV OCT images ($3.0\pm 0.7\;\text{plaques}\;\mathrm{per}\;{\mathrm{mm}}^{2}$). In the immunohistochemically stained sections, a considerably greater number of plaques ($66.0\pm 15.4\;\text{plaques}\;\mathrm{per}\;{\mathrm{mm}}^{2}$) were found compared with OCT and also compared with the Congo red-staining results ($2.0\pm 0.8\;\text{plaques}\;\mathrm{per}\;{\mathrm{mm}}^{2}$). Figure 6(f) shows the bar plot results for the plaque load evaluation in the five ROIs.

## 4 Discussion

A 1060-nm SS-OCT setup was used to investigate the feasibility of a 3D-printed tool to bridge the gap between OCT and histology and enable a direct one-to-one correlation. For this purpose, brains affected by AD were chosen as they are characterized by the deposition of extracellular amyloid-beta protein, which has been shown to possess hyperscattering properties when investigating with OCT.^18^^–^^23^ A-β plaques were identified as highly scattering structures in OCT images. These results are in good agreement with previously published work imaging A-β plaques using OCT.^18^^–^^23^ Previous work has shown that, depending on the used lateral and axial resolution of the setup, plaques of different sizes can be visualized.^18^^,^^20^^,^^21^^,^^23^ Using visible light and extended focus OCT setups, diffuse and neuritic plaques down to a diameter of 10  μm were investigated.^18^^,^^20^^,^^21^ However, using higher resolution sacrifices the imaging range. The presented setup has the big advantage that large FoV and high-resolution images can be acquired with perfect colocalization sequentially. The scan lens was chosen in order to provide a large FoV while still visualizing fine details such as A-β plaques in the small FoV. The large FoV image facilitates the direct correlation to histology, and the high-resolution modality reveals the brain morphology in micrometer resolution. A-β plaques are in the range of 10 to 200  μm in diameter in brain tissue.^2^^,^^3^^,^^8^ Using the high-resolution (lateral resolution of 4.8  μm) mode of this setup, plaques down to 20  μm could be visualized; see Figs. 5(h) and 6(c). Figures 5(h) and 6(c) show that dense (marked with blue arrows) and diffuse plaques (marked with red arrows) could be visualized using this setup. In the immunohistochemical stained section, a higher number of plaques were identified, in comparison with the high-resolution OCT ($6.0\pm 2.1\;\text{plaques}\;\mathrm{per}\;{\mathrm{mm}}^{2}$) and the large FoV OCT images ($3.0\pm 0.7\;\text{plaques}\;\mathrm{per}\;{\mathrm{mm}}^{2}$); see Fig. 6(f). This difference could be observed in the lower left corner in Figs. 5(h) and 5(i), respectively. A zoom-in of this region is shown in Fig. 5(j). Due to the lateral resolution of the OCT setup, particularly small plaques could not yet be resolved. An additional scan lens with a higher resolution could be implemented to overcome this limitation and resolve even smaller plaques.^21^^,^^24^ Using Congo red-stained histological sections, the A-β plaque load ($2.0\pm 0.8\;\text{plaques}\;\mathrm{per}\;{\mathrm{mm}}^{2}$) was shown to be lower compared with the plaque load found in immunohistochemical stained sections and even lower than the plaque load found in OCT images; see Fig. 6. These results are in accordance with the literature, and it is also described that multiple factors are responsible for this large plaque load difference, with resolution being one of the most important.^23^ The histological micrographs were acquired with a 40× commercial objective lens, providing a transverse resolution of 0.23  μm compared with 4.8  μm in OCM. Unfortunately, the image resolution of OCT is rather poor when compared with histology. As described by Gesperger et al., multiple single plaques in the immediate vicinity detected by histology might also be mistaken for a single, large plaque due to the limited resolution of our OCT setup.^23^ Therefore, our OCT setup might not be capable of visualizing all amyloid plaques. Further, the contrast in OCT images depends on the detected scattering signal, which in the case of the amyloid-beta plaques depends on the shape, size, and composition of these structures. In contrast, immunostaining is expected to pick up much fainter signals, making it possible to visualize amyloid-beta deposits of all different shapes and sizes very specifically. Our results for the investigated brain tissue show that OCT can detect neuritic and may to some extent also detect other types of amyloid-beta plaques. In the future, more samples from different brain regions, such as from the hippocampus and the temporal, frontal, and occipital cortex, shall be investigated and compared with brain maps to evaluate our method against state-of-the art tools for analyzing AD progression.^33^^,^^34^

To achieve an easy and direct correlation to histology, a tool consisting of two 3D-printed rings was designed. The used rings (until now limited to 35 mm in diameter) could be scaled to any size and were printed in a standard 3D printer (ULTIMAKER 3+). To achieve an even larger FoV, a different scan lens could be utilized. Alternatively, a translation stage could be implemented in the sample arm to acquire large mosaic images.^24^ These results could then be compared with histological images of whole brain slices.^35^ One limitation of the technique is that perfect correlation between high-resolution OCT, large FoV OCT images, and histological sections can only be achieved when areas with large fluctuations in the z-direction, for example, those found at vessel structures, were avoided. Our flexible approach could be used for the investigation of a variety of tissue types. Upon using histology, tissue shrinkage is unavoidable.^28^ In addition, in the sectioning process, artifacts can easily be introduced. Even for this work, the outer right part of the AD-affected brain tissue and the pons tissue in the upper left corner got detached while performing histology; see Figs. 4(a) and 5(a), respectively. To overcome this limitation and for a better direct correlation, the setup could be coupled to a vibratome to achieve improved correlation results.^36^^,^^37^

Using the presented SS-OCT instrument with the two FoV modalities, we were able to investigate AD-related pathology in *ex-vivo* human brain tissue. It was shown that higher wavelength regions show a reduced scattering and therefore improved penetration depth when imaging murine brain tissue.^38^ Our work showed that 1060-nm OCT was a good option to investigate AD brain tissue as the system was able to visualize the A-β plaques and at the same time the penetration depth was greater [see Fig. 5(d)] compared with shorter wavelengths.^20^^,^^21^ Using this setup, A-β plaques through 600  μm in depth could be identified. These plaques have been visualized using visible light, near-infrared, and light sources at 1300 nm.^18^^,^^20^^–^^23^ The present work shows that these hyperscattering structures can also be investigated using a 1060-nm OCT setup. The motivation of this work was to develop a flexible tool for label-free *ex-vivo* brain tissue investigations. In the future, this approach could easily be translated to *in-vivo* or *in-vitro* applications. Using a 1060-nm light source allows for imaging with deep penetration depths and still having a rather high axial resolution. The 3D-printed tool enabled a tissue preparation suitable for a one-to-one correlation to histology.

In the future, the SS-OCT system combined with further postprocessing steps such as differential phase contrast techniques might be a promising tool for performing stain-free, nondestructive, histology-like images in real-time in the field of neuropathology. As a next step, this approach could be used in a clinical setting where first quick overview images are acquired in the large FoV mode; afterward the clinicians could investigate interesting features using the high-resolution setting. This technique reduces the amount of data needed and enables a real-time and label-free investigation of brain tissue.

## 5 Conclusion

We demonstrated the use of a 1060-nm SS-OCT setup to perform sequentially large FoV and high-resolution small FoV imaging at the same position in unlabeled, *ex-vivo* AD-affected human brain tissue. With this procedure, we enabled an exact coregistration of OCT and histology data. In the future, this will allow for analyzing OCT data acquired in brain tissue in a more comprehensive manner. Further, the method is not restricted to brain tissue, but it could also be extended to any other tissue types in which it could be interesting to perform histology. Therefore, this versatile combination of our 3D-printed tool and OCT is a promising label-free, nondestructive approach for the field of neuroimaging.
